# Optimization of interpolation method for nitrate pollution in groundwater and assessing vulnerability with IPNOA and IPNOC method in Qazvin plain

**DOI:** 10.1186/s40201-017-0287-x

**Published:** 2017-11-21

**Authors:** Elham Kazemi, Hamid Karyab, Mohammad-Mehdi Emamjome

**Affiliations:** 1 Department of Environmental Health Engineering, School of Health, Qazvin University of Medical Sciences, Qazvin, Iran; 2 Bahonar Blvd, College of Medical Sciences, Qazvin, Iran

**Keywords:** Groundwater, Nitrate, Spatial interpolation, Vulnerability, IPNOA, IPNOC, Qazvin plain

## Abstract

**Background:**

The presence of nitrate is one of the factors limiting the quality of groundwater resources, particularly in arid and semi-arid climates. Therefore, the knowledge about the distribution of nitrate in groundwater and its source has an effective role in protecting health. The study aimed to optimize an interpolation method to predict the nitrate concentration and assessment of aquifer vulnerability in Qazvin plain.

**Methods:**

One hundred sixty-two deep wells in Qazvin plain aquifer were randomly selected and nitrate concentration was analyzed in four different lands including agricultural, residential, steppe and mixed-use areas. Interpolation was done by IDW, Spline, Kriging and National neighbor methods using ArcGIS software. To select the best interpolation method, errors of predicted values were determined by Mean Relative Error (RME) and Root Mean Square Error (RMSE). For analysis of potential vulnerability of aquifer to nitrate pollution due to agricultural activity and sewage leaks, hazard factors and control factors were used for identification of hazard indexes (HI) using IPNOA and IPNOC model.

**Results:**

The results showed that in 8.82% and 18.52% of samples in agricultural and residential areas, the detected nitrate was above the acceptable level at 50 mg/L. National neighbor method with the lowest RME and Spline method with the lowest RMSE were provided the most accurate estimates of nitrates in the aquifer. The highest hazard was obtained in agricultural areas (HI = 6.11). Also, the most influential parameters on aquifer vulnerability were mineral fertilizer (HF_f_ = 3), organic fertilizers (HF_m_ = 3), irrigation systems (CF_i_ = 1.04) and tillage patterns (CF_ap_ = 1.04).

**Conclusions:**

According to the results, National neighbor with the lowest RME was preferable than the other spatial interpolation methods for prediction of nitrate concentration in the aquifer. This method provided similar spatial distribution maps of nitrate in groundwater and that was an efficient method for assessing water quality. Hazard index as a result of agricultural activities (IPNOA) was ranged from “very low” to “low” which was in accordance with detected and predicted nitrate concentration in the aquifer. In addition he hazard of nitrate contamination from household (IPNOC) was in very low (class 2).

## Background

Due to its wide distribution and ease access, groundwater is considered as one of the most important sources of drinking water [1]. Thanks to high quality, these resources often do not require an advanced treatment [2]. But in recent years, various reasons including population growth, urbanization, heavy use of nitrogenous fertilizers in cropping agriculture systems [3, 4], not having a proper treatment for municipal and industrial wastewater have increased nitrate, nitrite and other chemicals concentration in groundwater [5–7]. Nitrogen is one of the most contaminations considered as a common problem in many parts of the world which can exist in two forms: organic and inorganic (including ammonium, ammonium compounds, nitrite and nitrate). Nitrates is considered as an important indicator of chemical in water and health, so that the World Health Organization has established a maximum contaminant level of 50 mg/L for drinking water [8]. Nitrate in drinking water can cause some serious diseases such as blue baby in infants and saliva, stomach, colon and bladder cancers in chronic exposures in adults [8–10].

Nowadays, due to increased human activities and negligence in chemical usage, an increase in nitrate concentration in residential areas groundwater seems natural. It can be attributed to the use of fertilizers, septic systems and leaking sewage systems [11, 12]. Many studies have shown high concentration of nitrate in areas with septic tank. It was illustrated that the groundwater resources were under strong anthropogenic pressure posed by the city [13]. Ouedraogo and Vanclooster (2016) showed nitrate problems in many metropolis in Africa [14]. In some cases, the increase in population has also considered as an influential factor in increasing nitrate concentration. Nas and Berktay showed that the average nitrate concentration increased from 2.2 to 16.1 mg/L during 1998 to 2001 in Konya, Turkey [15].

Today, efforts have been made to identify the predicting systems for water quality assessing as the best way to prevent pollution and investigate the quality of groundwater [16]. GIS (Geographic Information System) is considered as one of the most powerful technologies in this field to identify, analyze, interpret and make inferences about data [17]. Its capabilities for spatial interpolation have improved through integrating advanced methods as well as linking GIS to a system designed for modeling, analyzing, and visualizing a continuous field [18]. This system gathers data from a determined geographic location in order to store, collect and analyze data which is a great step to make a huge source of spatial and descriptive data accessible in a short time [9]. Numerous studies have shown that GIS used as explored spatial analyzes, interpolation and mapping all over the world. Also in science and health services, it can provide users and authorities with useful information [10, 19].

It is demonstrated that Kriging, IDW and Spline are efficient for spatial interpolation of nitrate concentrations in flat areas water resources [9, 18, 20]. Uyan and cay (2010) showed that Universal kriging, a type of geo-statistical technique, can be applied to distribute the groundwater nitrate concentration data [8]. In statistics, mainly in geo statistics, it is a powerful method of interpolation. The basic idea of Kriging is to predict the value of a function at a given point by computing a weighted average of the known values neighboring the function. Mathematically, the method is closely related to regression analysis. Semi-variogram plays a central role in the analysis of the geo statistical data using kriging method [21, 22]. It is showed that kriging method was the most suitable technique for mapping the bathymetry of the Yucatan submerged platform [23]. Spline method estimates values using a mathematical function that minimizes overall surface curvature. This results in a smooth surface passing exactly through the input point [24]. Inverse Distance Weight (IDW) is based on the extent of similarity of cells used in order to determine the depth and spatial variability of groundwater quality in areas which are not flat [17, 25, 26]. Azpurua and Dos Ramos showed that it is most likely to produce the best estimation in interpolation [27]. Natural Neighbor is based on a discrete set of spatial points. The value of an interpolation point is estimated using weighted values of the closest surrounding points in a triangulation [28]. One of the important keys in interpolation method is errors determination. There are a lot ways to determine the interpolation errors such as the Mean Bias Error and Root Mean Square Error [8, 17, 25]. The IPNOA is a parametric model which assesses the potential hazard of nitrate contamination originating from agriculture in aquifers. The method integrates the hazard factors (HF) and the control factors (CF). The HFs represents all farming activities that cause an impact on soil quality including application of fertilizers, livestock, poultry manure, industry wastewater and urban sludge. In addition the CFs assesses the characteristics of geographical location, climatic conditions and agronomic actions. IPNOA method has been used in several studies to assess the vulnerability of aquifers to nitrate [29–31].

In this article, after monitoring the nitrate concentration in selected groundwater resources, optimized spatial interpolation method and IPNOA index were implemented to present the groundwater vulnerability to nitrate in saline aquifer in Qazvin plain.

## Methods

### The study area and sampling stations

The research was done in Qazvin plain including two semi-arid and arid cold climates with an area of about 74,737 km^2^ in Qazvin province. This area was located in Saline Aquifer with an area of 9502 km^2^ in the northern basin. It was geographically situated between 49^°^ − 50^°^and 17^′^ − 32^′^at east longitude and 35^°^ − 36^°^and 39^′^ − 21^′^at north latitude including five cities, 14 sections, 30 districts, 18 towns, and 289 villages. The most important water resources in the study area were in Kharrud, a seasonal river, 3 permanent rivers, 189 springs and 23 aqueducts. In addition, more than 1200 semi-deep and deep wells were drilled to supply water for agricultural, industrial and residential destinations. The rain fall was 62 and 345 mm/year in the arid and semi-arid climates, respectively, with the average of 141 mm/year in the plain. Furthermore, the soil types in surface layers of arid climate and semi-arid climate were saline, sodium (alkaline) and gypsum.

**Fig. 1:**
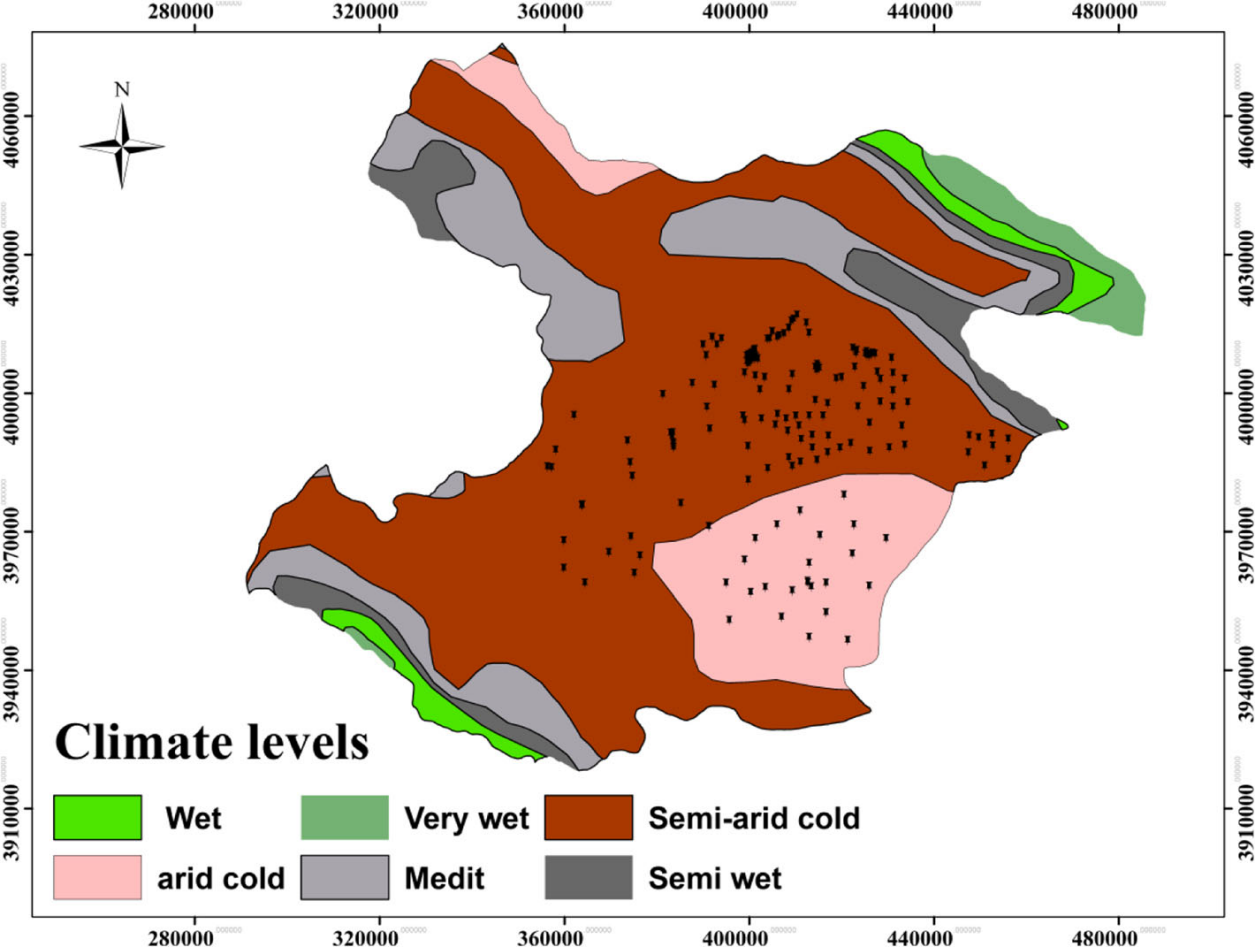
Position of sampling stations in the study area in Qazvin plain

**Fig. 2:**
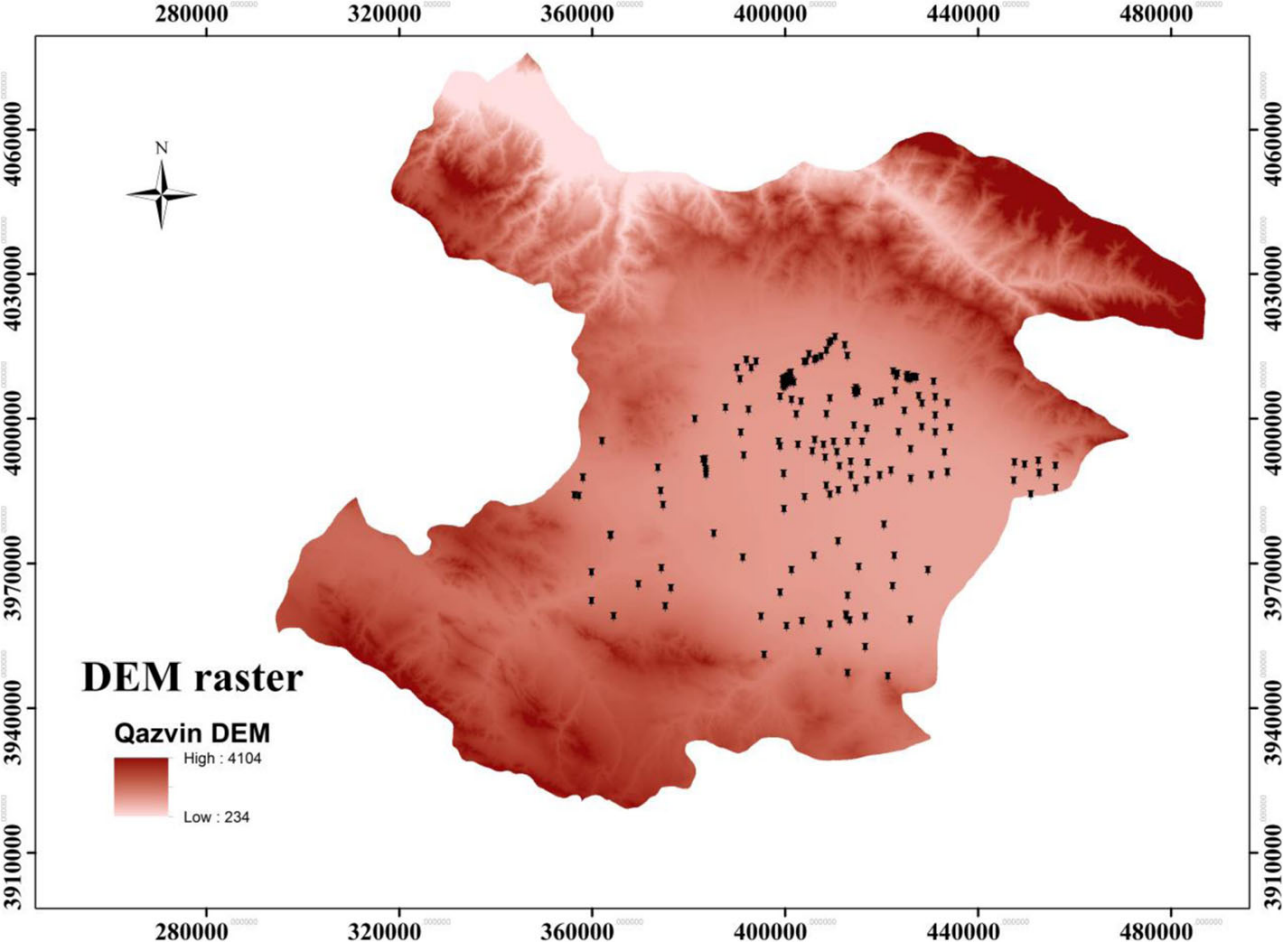
Position of sampling stations in Qazvin plain in DEM raster

Considering approximately 1000 underground wells, setting the confidence level at 95% and the average standard deviation at 1 mg/L, 162 wells were selected as the sample size in the arid and semi-arid climates. Different climates were specified by Dommartin method based on temperature and annual rainfall in the study area. In other studies, this categorization has been confirmed [32]. Since a vast area of the study was located in semi-arid region, the greater proportion of the samples was chosen from this climate. Figure 1 displayed the distribution of sampling points in different climates of the study area. As shown in Fig. 2, sampling stations were chosen in flat areas. In this figure, DEM (Digital Elevation Model) raster reveals that the study area was located at an altitude of 234 to 418 m above sea the level. Sampling stations were located in agricultural (41%), steppe (17%), residential (18%) and mixed- use (24%) areas. There was no station in industrial areas. Mixed-use was a type of area that blends residential, commercial, industrial and agricultural uses [33].

Calculated fixed radial (CFR) method based on saturated thickness (m), aquifer porosity (%), pumping rate (m^3^/year) and pumping time (year) was used to estimate groundwater quality protection zone [34]**.** Accordingly, it was approximately conducted 500 m around the water wells. Then, in this radius, factors affecting water pollution to nitrate including septic sewage discharges, fertilization, industrial activities, agronomic practices, irrigation systems and tillage were investigated by visiting around randomly selected water wells.

### Nitrate monitoring, quality control and data analysis

A cross-sectional study was done to monitor the nitrate concentration in the randomly selected groundwater resources in the study area during two seasons, spring and summer. In each stage, one liter water sample was taken from selected wells in a glass bottle. The grab samples were transported to a laboratory under controlled temperature conditions. Nitrate analysis was performed as soon as possible in accordance with the standard methods of water and wastewater examination [35] with a spectrophotometer DR6000 (HACH) at a wavelength of 220 nm.

In this study, precision, accuracy, representativeness and sensitivity were used as the quality control indicators. *Precision* was the degree of similarity among measurements taken from three repeated samples. It was calculated as the relative standard deviation (RSD). In general, data should be viewed with caution, when the RSD for triplicates is at 18%. It was calculated with Eq. 1, where s was standard deviation of the nitrate concentration [36].

1 $$ \mathrm{RSD}\kern0.5em \left(\%\right)=\frac{\mathrm{sd}}{\mathrm{mean}}\kern0.5em \times \kern0.5em 100 $$

*Accuracy* is an agreement measure for a variable value in a sample with a known or “true” value. In this study, spiked samples were used to provide an estimate of accuracy. Levels in the reference samples were selected within the range of values examined in the water body being assessed. For this purpose, certified reference samples of 5, 25 and 50 mg/l on nitrate with three repetitions were chosen to yield the accuracy. Finally, the results were presented as % of recovery using Eq. 2.

2 $$ \operatorname{Re}\operatorname{cov}\mathrm{ery}\kern0.5em \left(\%\right)=\frac{\mathrm{A}\hbox{-} \mathrm{B}}{\mathrm{A}}\times 100 $$

Where A was true value and B was measured nitrate concentration in the laboratory. The lab quality control samples must meet 85% - 115% recovery level. If the internal standard has a recovery of <70%, the results will be flagged and may be higher than what has been reported [37]. *Representativeness* is a qualitative term expressing how well the data which reflect the true environmental condition are sampled. It was a method to minimize the variability to ensure discriminate uniformity. It was visible in sample transportation and analyzing and using certified glass in lab [38]. *Sensitivity* is the capability of a method or instrument to discriminate between measurement responses for different levels of the variable of interest. It was investigated through detecting the minimum amount of nitrate concentration measured with spectrophotometer and considered 10 times lower than the water quality objectives for nitrate concentration in drinking water. Hallock and Ehinger (2003) express 20%, 7% and 0.01 mg/l, respectively, for accuracy, precision and detection limit in nitrate monitoring in water bodies [39–41].

Two sample t-tests using STATS.12 software was used for analysis of obtained data in arid and semi arid climates. In addition one-way analysis of variance was used for analysis of obtained data in four different land usages including agriculture, residential, steppe and mixed-use areas.

### Spatial interpolation and errors

GIS was used for interpolating the nitrate concentration using specific explanatory variables. Interpolation methods such as kiriging, spline, IDW and natural neighbor are powerful tools for data estimation based on the structure of a building [42]. Kriging assumed that the distance between sample points reflects a spatial correlation which can be used to explain variations. It is presented in Eq. 3. In this equation, F(x, y) were the estimated values of the index at the point with coordinates x and y. W_i_ was weight factor of points (i), and f_i_ was the index values in measuring points. In this equation, the amount of weight was provided through Variogram model [43].

3 $$ F\left(x,y\right)=\sum \limits_{i=1}^n{W}_i\times {f}_i $$

Inverse Distance Weighting (IDW) is a method to determine multivariate interpolation with a known scattered set of points. The assigned values to unknown points are calculated with a weighted average of the values available at the known points. In this method, the weight factor is determined based on points far from each other. Nearby points of samples were assigned with more weights. The IDW is presented in Eq. 4, where D_i_ was the proportion of the observed distance points to estimated point, π was potential distance weighting and n was the number of adjacent points. With the increasing power of the IDW, the amount of RMSE increases, as well [8].

4 $$ {y}_i=\frac{D^{-\pi }}{\sum_{i=1}^n{D}_i^{-\pi }} $$

The Spline method uses polynomials and is based on sample data fit a polynomial function from which values of unknown points are estimated. The key feature of spline is that there is no sudden change in level. According to the impact of degree to data interpolation, whether the higher degree is selected, the result will be smoother, but significantly the accuracy of the model is reduced. It is calculated by Eq. 5, where N was the number of sampling point, λ_j_was factor solution of linear equations and r_j_ was the distance from “j” sample points. Depending on the type of option T_(x, y)_, R(r_j_) was set by the user [44].

5 $$ {Z}_{\left(\mathrm{x},\mathrm{y}\right)}={\mathrm{T}}_{\left(\mathrm{x},\mathrm{y}\right)}+\sum \limits_{\mathrm{j}=1}^{\mathrm{N}}{\uplambda}_{\mathrm{j}}\;\mathrm{R}\left({\mathrm{r}}_{\mathrm{j}}\right) $$

Natural neighbor is another weighted-average method that has many positive features. It can be used for both interpolation and extrapolation. The basic equation used in natural neighbor interpolation is identical to the one used in IDW and can efficiently handle large input point databases [17]. In this study, to determine the interpolation errors, the values of RMSE, %RMSE and MRE were assessed by eqs 6, 7 and 8, respectively. The values of RMSE and MRE, the more approximating zero, indicate a better estimate of model used to assess the unknown parameters [2, 5].

6 $$ \mathrm{RMSE}=\sqrt{\frac{\sum_{j=1}^n{\left(x{(P)}_j-x{(m)}_j\right)}^2}{n}} $$

7 $$ \%\mathrm{RMSE}=\frac{RMSE}{\overline{X}}\times 100 $$

8 $$ MRE=\frac{1}{n}\sum \limits_{i=1}^n\left|\frac{z^{\ast}\left({x}_i\right)-z\left({x}_i\right)}{z\left({x}_i\right)}\right| $$

Where x(p) was the estimated value of each component, x(m) was the measurement of water quality component, n was sample number, $\overline{\mathrm{X}}$ was the average of a measured component, z(*x*_*i*_) was the observed value at location i, z*(*x*_*i*_) was the interpolated value at location i, and n was the sample size. Since RMSE is sensitive to outliers, %RMSE can be used to calculate the percent [45].

### Nitrate hazard index

Since 40.7% and 18.5% groundwater in the study were located in agriculture and residential areas, IPNOA and IPNOC were used to evaluate the hazard level of nitrate due to fertilization and sewage leakage. The IPNOC method assesses the Hazard Index from sewage leaks and sinkholes, whiles IPNOA gives a hazard potential from farming. Natural nitrogen content in soil, the climate, agronomic practices and the irrigation systems were assessed to obtain nitrate hazard index due to agricultural activities [28–30]. The relative hazard classes of different fertilization types and control factors are shown in Table 1. The hazard index (HI) for nitrate contamination was obtained by multiplying the different hazard factors (HF) by the control classes (CF) as shown in the Eq. 9. Where f was fertilizers, m was manure, s was sludge, n was soil nitrogen content, c was climate, ap was agronomic practices and i was irrigation system.

9 $$ HI=\left({HF}_f+{HF}_m+{HF}_s\right)\times {CF}_n\times {CF}_c\times {CF}_{ap}\times {CF}_i $$

**Table 1:** The hazard and control factors scores in assessment of vulnerability by IPNIA model

Relative hazard classes of different fertilization types									
Inorganic (kg/ha)		HF_f_	Organic (kg/ha)		HF_m_	Sludge (kg/ha)		HF_s_	
0		1	0		1	0		1	
1–25		2	1–150		2	1–150		2	
26–100		3	151–300		3	151–500		3	
100–180		4	301–500		4	501–1500		4	
>180		5	>500		5	>1500		5	
Control factors									
Soil nitrogen content (%)	CF_n_	Irrigation system	CF_i_	Rainfall (mm/year)	temperature (°C)	CF_c_	Tillage	Type of fertilization	CF_ap_
>0.5	1.04	Basin	1.06	>1200	6–15	1.01	Traditional	Fertirrigation	1.04
0.22–0.5	1.02	Border	1.04	1050–1150	13	1.08		Total surface	1.00
0.15–0.22	1.00	Sprinkler	1.02	950–1100	14–16	1.06		Through leaves	0.98
0.1–0.15	0.98	No irrigation	1.00	800–1000	12	1.04	Minimum	Localized	0.96
<0.1	0.96			600–1000	15–16	1.02	No tillage		0.94
				600–800	12–13	1.00			
				< 600	15–30	0.98			

Nitrogen leaking from the sewer pipes was assessed to obtain IPNOC index. Due to lack of information, nitrogen leakage from houses which not connected to sewerage systems was not considered. The annual value of nitrogen leaking from the sewer pipes (N_y_) was calculated with Eq. 10 which L_i_ was length of pipe (m), L_c_ was adjusted wastewater leakage (%) and N_i_ was average annual nitrogen load in transit (kg/year). The adjusted leakage percentage in each pipe (L_c_) was calculated based on its material and age using Eq. 11 which L_e_ was leakage related to the average life time of pipe (%).

10 $$ Ny=\sum {L}_c\times {N}_i\times {L}_i $$

11 $$ {L}_c={L}_e\left( age/ average\kern0.5em life\right) $$

Information required in this study was obtained from two sources. The first source was obtained from site visit and implementation of prepared checklist by researchers in the field including land-use, fertilizer types, fertilizer consumption, irrigation systems and tillage patterns in the study area. The second sources of information were obtained from government offices such as agriculture and water authority and meteorological organization. This information was included rainfall pattern, temperature, fertilization, soil nitrogen contents and general information of aquifer in the Qazvin plain. Finally hazard levels and vulnerability classification was obtained from Table 2. The prepared information from different sources were pipe material: concrete; average life time: 15 years; age of sewerage: 5 year; leakage: 7%; nitrogen production: 4.38 kg/capita.year; infiltration of nitrogen: 85%; number of inhabitants in each zone: 9812 person; area in each zone: 0.785 km^2^; mean of sewerage length: 21.23 m/ha; Rainfall: 62 mm/year in the arid and 345 mm/year in semi-arid climates; temperature: 13 °C; Soil nitrogen content: less than 0.1%; inorganic fertilization: 70–120 kg/ha in agricultural lands; Irrigation system: Sprinkler and border; Tillage: Traditional (no irrigation and no tillage was observed in steppe and residential area).

**Table 2:** Hazard index and relative classification [29]

Hazard index	Hazard level	Classification
2.54–3.18	1	Unlikely
3.19–5.88	2	Very low
5.89–7.42	3	low
7.43–9.31	4	Moderate
9.32–11.10	5	High
11.11–17.66	6	Very high

## Result and discussion

### Nitrate in different climates

As shown in Table 3, the recovery of triple samples was ranged in accepted levels of 85–115%. In addition, calculated RSD values were ranged between 0.01 to 0.04% that were lower than the acceptable value of 18%.

**Table 3:** The precision and accuracy of nitrate analysis in water samples

Concentration (mg/L)	n	Mean (mg/L)	Std. Dev.	RSD	Recovery	Std. Err. of mean	95% Conf. Interval of mean
5	3	5.64	0.02	0.01	113%	0.01	5.59–5.69
25	3	26.64	0.14	0.01	106.56%	0.08	26.29–26.99
50	3	51.65	2.03	0.04	103.3%	1.17	46.52–56.60

**Fig. 3:**
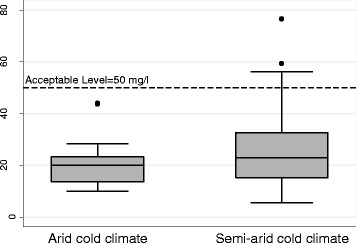
The distribution of nitrate in different climates in the study area

The means of nitrate concentration in semi-arid and arid cold climates were 25.66 and 19.97 mg/l (Table 4). The mean concentration of nitrate in semi-arid climate was 19.93% more than arid climate. In addition the results showed that nitrate concentration was significantly different in two climates (*p* < 0.05). The results indicated that nitrate concentration in both climates was significantly lower than acceptable level of 50 mg/l (p < 0.05). As presented in Fig. 3, the results showed that among 10 samples (4.6%) in semi-arid climate, the nitrate concentration was above the acceptable level of 50 mg/L in drinking water [46]. In addition, no unacceptable level was identified in water samples in arid climate. Williams and et al. (2016) in the assessment of California watershed concluded that nitrate in 42% of samples were highly more than the American standard. They concluded that nitrate was attributed to human activities [13].

**Table 4:** Nitrate concentration in water resources in different climates in Qazvin aquifer

Climates	n	Nitrate concentration (mg/L)				
		Range	mean	Std. Dev.	Std. Err. of mean	95% Conf. Interval of mean
Semi- arid	121	5.45–76.55	26.25	14.04	1.28	23.73–28.79
Arid	41	10.03–43.98	19.97	7.56	1.18	17.59–22.36
Total	162	5.45–76.55	24.66	12.98	1.02	22.64–26.68

### Nitrate in different land usages

The results of nitrate concentration in different land-use activities in the study area are shown in Table 5. The results showed that the mean of nitrate concentration has a significant difference in different land usages (F = 3.45, p < 0.05). The detected nitrate concentrations in different areas were ranked as mixed-use > residential > agricultural > steppe. In addition, in 8.82%, 18.52% and 20.08% of samples in agricultural, residential and mixed-use areas, the detected nitrate was above the acceptable level at 50 mg/L. The main reason for the nitrate detected in the residential areas was explained for the neighboring residential wells with cesspools. Also, the consumption of fertilizers in agricultural land was shown in studies as the main reason for the contamination of nitrate. The excessive use of nitrogen fertilizers not only increases groundwater pollution but also causes spatial spread of pollution. It is demonstrated that point and non-point sources, such as leaching chemical fertilizer in agriculture usage and leaching from septic and sewage discharges in residential usage can affect the nitrate concentration in groundwater [47]. Same results were reported by Schaider et al. (2016) which emphasized septic systems were as major sources for the contamination of aquifer with various pollutants such as nitrate [48]. Inverse relation between fertilizers application rate and groundwater nitrate concentration was reported by Mahvi et al. (2005). They explained that the reason was soil characteristics in different regions [49].

**Table 5:** Nitrate concentration in water resources in different land usages in Qazvin aquifer

Land usages	n	Range	mean	Std. Dev.		
Agricultural	66	5.45–76.55	22.45	9.51		
Steppe	27	9.79–32.56	21.05	11.64		
Residential	30	6.67–56.17	26.58	15.44		
Mixed-use	39	8.30–76.55	29.43	15.54		
Source	SS	Df	MS	F	Prob > F	Prob > chi2
Between groups	1670.22	3	556.74	3.45	0.018	0.002

The results showed that 27 sample stations were located in residential areas at a distance of 500 m between water wells and residential septic based on CFR method. According to the obtain results, it can be concluded that sample stations located in residential area were more contaminated than agricultural areas. Because the different density of settlements around water wells and lack of access to precision distance between water wells and septic systems in residential land-use, relationship between population density and nitrate concentration in aquifer could not be possible. Schneider et al. (2016) showed that septic systems were a major source of nitrate in groundwater; however, they could not show a significant relationship between density and nitrate concentration in aquifer [48]. The obtained results did not correspond with the study carried out by Kristin et al. (2005) that introduced agricultural land-uses as the main reason for the groundwater contamination in Nantucket Island, in Massachusetts, America [15]. These results emphasize that the contamination of water resources was caused by man-made factors more than natural factors in Qazvin plain. In site visits, it was revealed that cesspools were the major man-made factor that can affect water quality in residential lands in Qazvin plain.

Agricultural activities were an important factor affecting the quality of water resource, especially in arid and semi-arid areas in the study area, in the same line, more than 40% of sample stations which were located in the agricultural lands had the mean nitrate concentration of 25.27 mg/L. Too much agricultural activities, using fertilizers especially nitrogen ones and high pumping of groundwater were the most effective factors observed in the site visit in the study area. In other studies, the role of agricultural activities in the contamination of groundwater with nitrates was proven (Jafari Malak Abadi. 2002). The highest nitrate concentrations were detected in mixed-use land, which blends residential, commercial, industrial and agricultural uses. Increasing nitrate concentration in this area can be attributed to the synergistic agents. Also, some studies have showed that industrial activities may highly increase nitrate concentration in groundwater resources [50]. The lowest concentration of nitrates was identified in steppe land. This can be due lack of human activities, the soil type and properties such as texture, structure, rainfall, irrigation, evaporation and transpiration [51].

### Spatial interpolation of nitrate

**Fig. 4:**
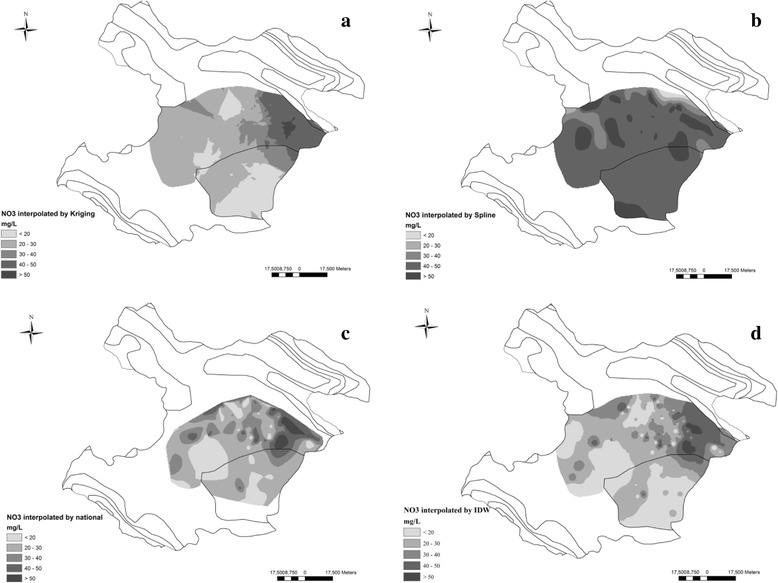
Interpolation of nitrate concentration (mg/L) in Qazvin plain prepared using Kriging (**a**), Spline (**b**), National neighbor (**c**) and IDW (**d**) methods

Figure 4 shows the spatial distribution of nitrate concentration in the study area. Patterns a, b, c and d were obtained by interpolating the satisfaction levels using Kriging, Spline, Natural Neighbor and IDW methods, respectively. Their outputs showed that in most of the study areas nitrate concentration was variable between 20 to 30 mg/L. One of the problems in spatial interpolation is errors that can derivate from estimation of unknown values. As presented in Table 6, cross validation in results showed that the interpolation by National Neighbor had the lowest amount of MRE, while the Spline method presented the lowest RMSE. The obtained results were not consistent with other studies that emphasized the kriging method was the most suitable technique for mapping [23]. Acceptable amount for errors is not provided because it depends on the quality of the map being geo-referenced, the quality of the target (base) map and the purpose of the geo referencing.

**Table 6:** The prediction accuracy of interpolation methods

Methods	Mean relative error	Root mean square error	% RMSE
Kriging	0.21	11.93	0.483
Natural neighbor	0.01	12.47	0.505
IDW	0.10	28.49	1.155
Spline	0.21	5.25	0.212

### Potential risk of nitrate

The obtained data from inorganic and organic fertilizers (kg/ha), sludge usage (kg/ha), Soil nitrogen content (%), irrigation systems, rainfall (mm/year), temperature (°C) and tillage patterns were converted into scores including HF_f_, HF_m_, HF_s_, CF_n_, CF_i_, CF_c_, CF_ap_ and HI_i_. As presented in Table 7, the highest nitrate hazard due to farming and fertilization was obtained in agricultural areas with HI of 6.11 whereas HI of nitrogen leaking from sewerage in residential areas was 7.12. Obtained results showed that hazard index, as a result of agricultural activities, was ranged from “very low” to “low” which was in accordance with detected and interpolated nitrate concentration in the aquifer. In addition he hazard of nitrate contamination from household (IPNOC) was in very low (class 2). In assessment of IPNOA index, the maximum aquifer vulnerability was appertained to mineral fertilizer (HF_f_ = 3), whereas sewage sludge have no major effect on hazard levels (HF_m_ = 1). Despite of limitations in considering soil characteristics and hydrological structure of subsoil, IPNOA and IPNOC methods can give an evaluation of potential risk of groundwater contamination.

**Table 7:** Average of hazard indexes using IPNOA method in the study area

Land usage	HI_i_	Hazard level	Hazard classification
Agricultural	6.11	3	Low
Steppe	3.05	2	Very low
Residential	3.05	2	Very low
Mixed	5.09	2	Very low
Average	4.08	2	Very low

## Conclusion

The research was developed to monitoring and optimization an interpolation method to predict the nitrate concentration and assessment of aquifer vulnerability. The mean concentration of nitrate in semi-arid climate was 19.93% more than arid climate and in 4.6% samples the detected nitrate was above the acceptable level of 50 mg/L. In addition the results showed that nitrate concentration was significantly different in different climates and land usages (*p* < 0.05). In addition in 8.82%, 18.52% and 20.08% of samples in agricultural, residential and mixed-use areas, the detected nitrate was above the acceptable level at 50 mg/L. Application of IDW, kriging, National Neighbor and Spline methods represents that National neighbor with the lowest RME and Spline with the lowest RMSE provide the most accurate estimates of nitrates in the aquifer. The results of parametric models of IPNOA and IPNOC showed that the quality of Qazvin aquifer is mainly influenced by the fertilization in agriculture land and wastewater leakage in residential areas. Obtained results emphasize that conserving practices are highly important since the groundwater resources are limited in arid and semi-arid climates in aquifer of Qazvin plain.

## Abbreviations

CF: control factor
GIS: Geographic Information System
HF: hazard factor
HI: hazard index
IDW: Inverse Distance Weighting
RME: Mean Relative Error
RMSE: Root Mean Square Error
RSD: relative standard deviation
